# The Changing Role of Chinese English-as-Foreign-Language Teachers in the Context of Curriculum Reform: Teachers’ Understanding of Their New Role

**DOI:** 10.3389/fpsyg.2022.904071

**Published:** 2022-07-06

**Authors:** Man Lei, Jane Medwell

**Affiliations:** ^1^Foreign Languages College, Shanghai Normal University, Shanghai, China; ^2^School of Education, University of Nottingham, Nottingham, United Kingdom

**Keywords:** teacher’s role, English-as-foreign language teachers, curriculum reform, China, humanistic values, teacher reflection

## Abstract

The New Curriculum Standards for teaching English introduced major changes in the culture of teaching and learning English in the Peoples Republic of China (PRC). Changes have been linked to changing goals for English instruction and a revision of Confucian values in schooling. In this article, we argue that this English curriculum proposes a new role, with new demands, for English-as-foreign-language (EFL) teachers in the PRC. In order to implement the curriculum reform successfully, teachers involved in the reform are required to have a shared understanding of its nature, purposes and scope. However, little is known about to what extent EFL teachers understand and engage with their new roles. This study examines teachers’ understandings of the new curriculum and of the new, demanding role of teachers implied by the curriculum. This is a mixed methods study involving an analysis of the curriculum document, a survey (*n* = 227) of EFL teachers and semi-structured interviews with a sample of teachers in the cohort (*n* = 18). The findings suggest that many teachers know the content of the curriculum document, but expressed uncertainty about the implications of changes, uncertainty about what a shift to student-centered teaching and learning means and confusion about new professional development demands. The findings of this study have wider implications for EFL teachers, teacher educators, researchers and policy makers in the PRC and similar national contexts. This article highlights that, from an international perspective, introducing new ideas and practices should consider teachers’ existing understanding and experiences of the curriculum as well as the way in which they understand the purposes of the changes, and should promote a shared understanding of policy intentions.

## Introduction

Curriculum reform is seen as a dynamic and complex reality in teachers’ professional lives (Vähäsantanen, 2015). EFL teachers are required to constantly develop their professionalism to better serve the goals of curriculum reform (Jiang and Zhang, 2021). Professional development of EFL teachers, however, involves not only continuous learning but also the cultivation of teachers’ new roles (Vähäsantanen and Eteläpelto, 2009; Yang, 2015; Tao and Gao, 2017; Jiang, 2022; Lei and Xu, 2022). The New Curriculum Standards (NCS) for teaching English in senior high schools (Ministry of Education [MOE], 2018) in the PRC were built upon 15 years of piloting. The NCS document proposes a new relationship between teachers and students, based on the centrality of Confucian humanistic values, to harness students’ emotions and feelings so that they can take a more active role in learning English and engage in more communicative activities. To achieve this, the NCS demands significant changes in teachers’ role, teaching practices, and their focus on students. Moreover, the NCS is open to interpretation in ways its predecessors were not, calling for teachers to be creative within the curriculum and to make curriculum choices (Mei, 2019). This raises the question of how well teachers understand and engage with their new roles. In order to implement a curriculum reform successfully, teachers involved are required to have a common understanding of its nature, purposes and scope (Vähäsantanen and Eteläpelto, 2009; Fullan, 2012; Yang, 2015; Lei and Medwell, 2020; Greenier et al., 2021; Lei, 2022). The present article reports the findings of an analysis of the NCS document focusing on two of the key areas of change proposed—the teacher’s role and humanistic values. The paper then reports on a study of how teachers understand their new roles and the demands of the NCS, understandings which are at the core of enabling more than one million EFL teachers in China’s high school education system to enhance their professionalism and empowerment, and to develop new and demanding approach to teaching.

## Teachers’ Understanding of Educational Reform

Teachers’ understandings of the key concepts of a change and their background training have a great impact on the implementation of an educational reform (Kırkgöz, 2008; Vähäsantanen, 2015; Tao and Gao, 2017). Researchers argue that if teachers are to successfully implement an educational reform, they must have a common understanding of the theoretical principles and classroom applications of the changes proposed by the reform (Vähäsantanen and Eteläpelto, 2009; Fullan, 2012; Yang, 2015; Lei and Medwell, 2020). Of the two, the latter often proves to be the most important, especially when teachers are poorly trained or lack sound knowledge of the educational reform. In the context of curriculum reform, it is very necessary and important to change the way teachers think about key components of an innovation, which is a more complex, inner, and implicit change (Fullan, 2012; Vähäsantanen, 2015). This emphasizes that the change in teachers’ understandings and beliefs plays a significant role in the curriculum reform.

### Teacher Training for the New Curriculum Standards

The NCS demands that EFL teachers improve their levels of professionalism through continuous learning in a number of areas and demands that they should constantly update their language knowledge and proficiency in order to be good teachers in a modern society (Ministry of Education [MOE], 2018). Teacher training plays an important role in how far curriculum changes can be successfully implemented (Carless, 1998). Kırkgöz (2008) claims that teachers need guidance and opportunities to learn the new content and methods of communicating with learners, otherwise educational reform cannot be implemented successfully.

Chinese teachers’ views of teaching are likely to be influenced by traditional teaching concepts (Jin and Cortazzi, 2006). Teacher training programs, therefore, ought to be capable of updating teachers’ knowledge and of bringing about large-scale changes in teachers’ existing beliefs to increase their awareness of the changes in the NCS in order to help them adapt to the innovation. However, teacher training in China is delivered mainly through short intensive courses attended by teachers on a selection basis and thus may not support all teachers. Even if short courses have a huge impact on some teachers, these may, without proper guidance, have difficulty understanding the new concepts or fall back on their previous teaching experiences and ignore the innovations (Fullan, 2012).

Moreover, as Ping (2010) pointed out, there is an identified problem for teacher trainers in China, in that their training programs tend to lack interactions, with trainees tending to be unresponsive. Ping (2010) describes such training as involving mainly passive classes with largely silent learners. This has been a frustrating experience for both trainers and trainees, leading to some unexpected results. For example, passive training is generally not stimulating or interesting for trainees; trainees may ignore the value of the things they have learned during the training program because of lack of motivation; the trainer may lack enthusiasm and energy when teaching the class; trainees may not understand the training content well because they tend not to interrupt the trainer with their questions. Such a passive learning approach may be the result of two features. The most important of these is the highly hierarchical nature of Chinese culture, which lowers learners’ status and leads them to be passive recipients to receive whatever their higher status teachers transmit to them. The second reason is that questions or challenges from learners may put teachers at the risk of losing face because they may not have the correct answers (Biggs, 1996; Zheng, 2013; Li and Wegerif, 2014; Lei, 2022).

### Relationship Between Teachers’ Beliefs and Practices

Understanding the beliefs of teachers and their effects on teaching may be a key feature of the success of educational reforms (Fullan, 2012). Brown and Cooney (1982) defined beliefs as the key determining factors of an individual’s action which guide their behavior. Teachers’ beliefs about English language teaching (including teachers’ understandings, attitudes, expectations, values, and theories about teaching and learning) are normally affected by their previous experience as learners at school; their experience as classroom observers; their teaching experience; their prior training experience (Vähäsantanen and Eteläpelto, 2009; Vähäsantanen, 2015; Yang, 2015).

Researchers have varied views on the relationship between teachers’ beliefs and their classroom practices. Some studies suggest a consistent relationship between teachers’ beliefs and practices, e.g., Pajares (1992) refers to beliefs as *messy constructs* but argues that there is a close relationship between beliefs and knowledge. He points out that teachers’ beliefs are far more influential than their knowledge on the way they organize tasks and solve problems, on the kinds of decisions they make, on the way they plan lessons, and on the way they behave in the classroom. Many researchers (e.g., Burns, 1992; Breen et al., 2001; Gu, 2009; Fullan, 2012) have a similar view about the importance of teachers’ beliefs for EFL classroom practice, showing that teachers’ practices tend to be highly consistent with their beliefs. EFL teachers bring their own beliefs to situations related to English teaching, and their beliefs are normally regarded as important predictors of their general classroom practice. Their concepts of teaching reflect their beliefs about teaching, affecting their understanding and attitudes and also guiding their behavior.

Teaching methods encapsulate the way teachers put their beliefs into classroom practice. Therefore, it is necessary to understand teachers’ beliefs in order to design any professional development program that aims to change classroom practices (Medwell et al., 1999; Fullan, 2012). In the case of curriculum reform, Kennedy (1988, p. 329) suggests, “teachers may be required to change the way they think about certain issues, which is a deeper and more complex change.” That is to say, it may be necessary and important to change teachers’ beliefs in order to implement any educational reform.

However, some studies suggest that changes in teachers’ beliefs, understandings, and attitudes are likely to follow changes in their behavior rather than determine it. For example, Huberman’s (1981) study of a reading program innovation showed that initial teacher training and ongoing guidance needed to be provided for teachers to help them adapt to an innovation. All the teachers, trainers and administrators in that study suffered a period of high confusion and anxiety because of the introduction of the new program. According to Huberman (1981), after the new program started, the teachers still needed some time to link their behavior with the concepts of the program. Even 6 months later, the teachers still had little sense of why specific behavior patterns could lead to certain results.

Some research also indicates that there may be inconsistencies between teachers’ beliefs and their observed practices (e.g., Basturkmen et al., 2004; Farrell and Lim, 2005) and several studies have even found no significant correlation between the two (e.g., Yim, 1993). This may be because there are many other factors that can greatly influence teachers’ beliefs during their actual classroom practice (e.g., Farrell and Lim, 2005; Fullan, 2012). For example, there may be inconsistencies between beliefs and practices if the teacher is in the process of coping with changes in his/her beliefs before putting changes into actual practice, when some propositions are incompatible, or when there are multiple belief systems (Graden, 1996). Moreover, it has been suggested that different research methods can affect whether the findings indicate limited consistencies between teachers’ beliefs and their practices (Basturkmen, 2012), but sophisticated methods do not necessarily indicate a high degree of correspondence either.

Indeed, the relationship between teachers’ beliefs and their practices is complicated (Pajares, 1992); it can be described as *dialectical* rather than *unilateral*. Beliefs and practices can affect each other, i.e., beliefs can guide and shape behavior but reflections on experiences and behavior can influence (and possibly change) beliefs (Breen et al., 2001). The study reported here takes teachers’ experiences into consideration when examining teachers’ perceptions and needs in relation to the NCS.

## An Overview of the New Curriculum Standards

The NCS proposes some very significant changes to the role of EFL teachers in resource planning, classroom teaching, choice making, and approach toward students rather than demanding that teachers should strictly comply with textbook-based, top-down curriculum delivery. In contrast to previous attempts to introduce communicative language teaching, which have been criticized as imposing western approaches in China (Hu, 2005), the NCS does put more emphasis on the tenets of Confucianism, of mutual respect and care (Ministry of Education [MOE], 2018; Cheng and Zhang, 2020). The “figure of Confucius has been a key feature of revisionist assessments” of education (Lei, 2020, p. 174), and Confucius still plays an important role as a key cultural philosopher of the modern PRC, influencing education in China and other cultures, particularly the student-teacher relationship (Cheng, 2017). Confucius set a lofty example for all teachers to emulate, and is often referred to as “an exemplary teacher for all ages” and “the greatest sage and teacher” (Rao, 1998, p. 49). From his own teaching practice, Confucius proposed the requirements for being a good teacher in Chinese culture and these values still have a very important influence on understandings about the role of teachers in modern East Asian societies, which are often referred to as *Confucian Heritage Cultures* (Biggs, 1996; Yu, 2011; Li and Wegerif, 2014). Until quite recently Confucius’ legacy was interpreted in ways that positioned teachers as holders of all knowledge, the center of the classroom and unquestionable (Hu, 2002; Cottine, 2016). English teaching across the PRC, in the past, was teacher-centered and teacher-dominated (Cortazzi and Jin, 1996; Jin and Cortazzi, 2006; Zheng, 2013; Lei and Medwell, 2020) by teachers who were good models, holders of profound knowledge and in unquestionable command of the class. However, “*good teacher”* is a cultural construct which can change over time and the NCS signals a massive challenge to the traditional Chinese construct of a good teacher. The underpinning for this, Confucianism, has become more popular in the past two decades in the PRC (Deng and Smith, 2018) and has interpreted the role of the teacher rather differently, with the focus placed more on the development of students’ “humanistic values” by their teachers. To achieve this, the “good teacher” presented in the NCS has features of the teacher as guide, organizer, reflective teacher, rather than merely the dominant actor in the classroom. The EFL teacher is seen as engaged in a collective enterprise of learning with the students (Ministry of Education [MOE], 2018), rather than being only an authoritative exemplar. This new vision of the teacher and teaching is based, at least in part, on the promotion of humanistic values in the NCS, a term which has strong Confucian resonances and is used many times in the NCS document. “Humanistic values” is clearly explicated in the NCS as “learning English for not only economic purposes – but also for the cultivation of students’ positive virtues and personality traits, and the development of their world-views, in individuals and the society to which they belong” (Ministry of Education [MOE], 2018, p. 3). This demands EFL teachers consider students’ concerns, feelings, in order to inspire them to form positive attitudes to learning and develop a healthy personality (Mei and Wang, 2018; Ministry of Education [MOE], 2018). Humanistic values also refer to another aspect of the Confucian tradition of valuing “humanity,” which refers to the support and care for one’s students and developing a Confucian concern for the harmony of the students, class and community (Cheng and Zhang, 2020).

As Hu (2002, p. 98) noted, “a fundamental assumption of Confucian tradition is that innate ability does not account for success or failure in education, and there is a strong belief that everyone is educable and capable of attaining perfection.” However, in the past, this has led to an emphasis on the responsibilities of students to study diligently. The NCS shifts responsibility for student progress to the teachers’ actions. EFL teachers are required to motivate all the students, prompt their positive attitudes in English learning process, and help all the students get of continuous progress and development (Ministry of Education [MOE], 2018). The 2003 curriculum document required EFL teachers to provide students with opportunities to facilitate their independent learning and learner autonomy. However, the expected teacher role documented in the NCS goes much further by demanding that teachers guide their students to participate in the learning process more actively through various collaborative work and help students to build their confidence within this collaborative learning process (Ministry of Education [MOE], 2018). The curriculum provides teachers with explicit examples to show them how to guide students in co-operative learning. Furthermore, the NCS suggests teachers should be aware of their responsibility to prompt learners’ willingness to cooperate with peers. This student-centered approach of taking account of students’ feelings, guiding and helping students through planning particular lessons and differentiating content may be a very new and different role to teachers who are accustomed to straightforward correction but it can be traced back to a Confucian emphasis on inculcating humanistic values and harmony in students.

Another area where the NCS seemed likely to challenge teachers’ existing practices and beliefs is in the view of how they evaluate their teaching. The NCS places much greater emphasis on EFL teachers reflecting on their teaching—what went well, not so well and so on—than the earlier approach, by illustrating, in detail, the importance and benefits of teacher reflection and inquiry. Reflection can help teachers to “identify and solve problems in professional work” (Ministry of Education [MOE], 2018, p. 28), so this is beneficial for teachers’ professional growth and development. But most importantly, teachers will undertake reflection not individually, but within peer groups to address the challenges and problems facing them in their day-to-day professional lives. This emphasis on reflection suggests it can become a mechanism for developing teachers’ knowledge and understanding, and help them to adapt to the changes in their role.

The NCS encourages teachers to create a culture of cooperative learning with other teachers and cooperative inquiry that encourages communication, sharing with colleagues, and cooperative exploration (Ministry of Education [MOE], 2018). This was not included in the 2003 curriculum—the suggestion that teachers should work together as a learning community to prompt each other’s professional development and enhance their understanding and awareness of new role. This integration of teacher communication and reflection to prompt teacher professionalism has been explored in literature (e.g., Lai, 2010; Lei and Medwell, 2020) and underpinned projects like “lesson study-based action education” (Gu and Wang, 2003) to scaffold teacher learning within community. This view of teacher professionalism was a very significant change for teachers to take on.

## Challenges Posed by the New Curriculum Standards for English-as-Foreign-Language Teachers

The challenges posed by the NCS for EFL teachers can be summarized as three Cs – clarity, complexity, context (Fullan, 2007; Kırkgöz, 2008; Vähäsantanen and Eteläpelto, 2009).

Clarity here means whether teachers are able to identify essential features of the change (Fullan, 2012). Lack of clarity may refer to unclear objectives or unspecified ways of implementation. Even when the need for an innovation has been recognized, pinpointing what teachers need to do differently is always a barrier to the change process. Fullan (2012) pointed out that, in order to achieve clarity, the involved individuals need a sense of purpose that is explicit, shared, flexible, as they are required to adapt to changing circumstances constantly. However, explicit purposes are absent in much curriculum change documentation. Kırkgöz, 2008 found that most teachers in their study were unable to identify the main and key features of the curriculum. Lack of clarity of the curriculum, therefore, represents a major problem during the implementation phase. In our review of the NCS document, however, we found it did clarify its expected goals and gave suggestions and teaching examples to guide EFL teachers. This raised the question of whether the teachers in our study were able to identify the essential features of the NCS.

Complexity refers to the difficulty of and the extent to which practitioners are responsible for, implementation (Fullan, 2012). The complexity of an educational innovation has been discussed by many researchers (Carless, 1998; Vähäsantanen and Eteläpelto, 2009). Generally speaking, the nature of change is multidimensional and takes place in a particular context that includes political, social, economic and moral aspects. The organizations and individuals involved as well as the particular contexts are just a few of the factors in any change effort. More specifically, concerning the actual components or dimensions of an innovation, the level of complexity mainly depends on the new materials, new teaching strategies and alteration of beliefs (Basturkmen et al., 2004; Fullan, 2012). According to Fullan and Pomfret (1977), changes that provide a certain level of complexity, but not to the extent that the level of adjustment required by teachers becomes overwhelming, are more likely to be implemented effectively in practice. However, changes that are incompatible with teachers’ existing beliefs and strategies, and impractical or unpiloted are more likely to pose challenges to implementing the change (Carless, 1998). For example, changes in the use of new materials without any other changes in strategies may be a minor change. If a change includes other aspects, it tends to be rather more complex. The complexity of a change is not only a feature of the change itself but also a feature of teachers in terms of the discrepancy between the teacher’s current practice and beliefs, and those assumed by the change. In the case of the NCS reform, the assumed changes are not just to materials but also to aspects such as teaching practices and beliefs. So, the NCS is not a minor change but a more complex one.

In this study, although the educational change is “top-down,” it is impossible to say that the NCS is only curriculum change, because it calls for a change in teacher behavior and beliefs. The Ministry of Education published the NCS to change the experience of students and their learning outcomes, to solve the problems of teaching in practice and to cope with the difficulties of teacher development. This revised curriculum includes profound changes both to curriculum content and the role of the teacher (Ministry of Education [MOE], 2018). However, to activate the changes required by the NCS, teachers have to teach differently and need to know different things. Teachers may need to change their attitudes, views and teaching behaviors in order to meet the NCS’ requirements. Because of the perceived dominant role of teachers in the classroom in a traditional Chinese teaching context and the influence of teachers’ beliefs and experience, it could be difficult and challenging for teachers to adopt the new roles advocated in the NCS which threaten their authority in the classroom.

Analysis of the NCS exposes a fundamentally different approach to the curriculum and the teacher’s role in it. The NCS offers a transformative view of what teachers do, implicitly requiring EFL teachers not just to change their teaching practices, but also the ways they learn about teaching. They are expected to adopt a more communicative approach and to develop their own professional knowledge through reflection and communication. Chinese EFL teachers whose training and professional practice comes from past traditions, might find the NCS unfamiliar and very challenging when required to adapt from being “good teachers” in the traditional context to “good teachers” in the modern society (Zhu, 2018), and it is not known what teachers understand about their new roles.

## Methods of This Study

The research reported here is a part of a larger research project (between 2018 and 2021) addressing one key research question:

• How do EFL teachers understand their new roles and the demands of teaching the NCS?

The present study involved the administration of a questionnaire to 273 EFL teachers and subsequent semi-structured interviews with a sample of teachers within the cohort. Official approval from the collaborators’ universities was sought before carrying out the study involving the teachers.

The questionnaire comprised two sections. The first section of the questionnaire collected the demographic data of the respondents. The second section asked teachers about the extent to which they knew about the key components of the NCS in relation to humanistic values, new roles (as organizer, guide, and reflective teacher), the requirements of the NCS (student-centered teaching and differentiation) as specified in the document. The questions in the questionnaire took the form of Likert Scale type responses to 28 statements about their understanding of their new role and their teaching practices (Tables 1,– 3). All the respondents answered the questionnaire anonymously and voluntarily. To make sure that the participants could understand the questionnaire, the items and format were piloted and revised with 24 EFL teachers (not included in the main study). The revised questionnaire was then distributed to EFL teachers in a prefecture-level city in Southern China. Cluster sampling was adopted to select the teachers and schools within this geographical location for the questionnaire. 273 questionnaires were distributed generating 227 returns, a very high return rate of 83%. The questionnaire sample had more female teachers (90%) than male teachers (10%). This is a reflection of female dominance in EFL teaching in the PRC (Beijing Normal University, 2013). The teaching experience of the teachers responding to the questionnaire ranged from 2 to 32 years in middle schools (Table 4).

**TABLE 1:** Teachers’ responses to statements about humanistic values.

Statements	Strongly disagree	Disagree	Neither agree nor disagree	Agree	Strongly agree
					
	n (%)	n (%)	n (%)	n (%)	n (%)
1. EFL teaching is not for not only economic purposes – but also for the cultivation of students’ positive virtues and personality traits, and the development of their world-views	4 (2)	6 (3)	50 (22)	133 (58)	34 (15)
2. The NCS defines the nature of the English courses as the combination of humanistic and instrumental values	4 (2)	9 (4)	49 (17)	146 (64)	29 (13)
3. Teaching English to develop students’ positive virtues and personality traits, and the development of their world-views is successful in practice	3 (1)	24 (11)	79 (35)	111 (49)	17 (7)
4. Teaching English for the cultivation of students’ positive virtues and personality traits, and the development of their world-views is difficult	8 (3)	34 (15)	33 (14)	139 (62)	13 (6)

**TABLE 2:** Teachers’ responses to statements about teacher role.

Statements	Strongly disagree	Disagree	Neither agree nor disagree	Agree	Strongly agree
					
	n (%)	n (%)	n (%)	n (%)	n (%)
5. English classes should be student-centered. Teachers should not dominate the class	4 (2)	6 (3)	15 (7)	140 (61)	62 (27)
6. Student-centered teaching is successful in practice	6 (3)	22 (10)	81 (35)	109 (48)	9 (4)
7. Making students as the center of the class is difficult	12 (5)	55 (24)	57 (25)	91 (41)	12 (5)
8. The EFL teacher’s role is only to teach knowledge of foreign languages	43 (19)	118 (52)	21 (9)	39 (17)	6 (3)
9. The NCS emphasizes that EFL teachers should be the center of the class	53 (23)	157 (46)	27 (12)	36 (16)	8 (3)
10. EFL teachers should set realistic teaching objectives according to local teaching needs and students’ language proficiency	4 (2)	9 (4)	11 (5)	105 (46)	98 (43)
11. The NCS emphasizes that EFL teachers should set realistic teaching objectives according to local teaching needs and students’ language proficiency	5 (2)	3 (1)	21 (9)	151 (67)	60 (21)
12. Setting realistic teaching objectives according to local teaching needs and students’ language proficiency is successful in practice	4 (2)	19 (8)	59 (26)	131 (58)	14 (6)
13. Setting realistic teaching objectives according to local teaching needs and students’ language proficiency is difficult	6 (2)	49 (22)	33 (14)	117 (52)	22 (10)
14. EFL teachers should plan different resources and teaching methods according to students’ different situations	4 (2)	8 (4)	18 (8)	85 (53)	75 (33)
15. EFL teachers should plan different resources and teaching methods according to students’ different situations	4 (2)	8 (4)	18 (8)	85 (53)	75 (33)
16. The NCS emphasizes that EFL teachers should plan different resources and teaching methods according to students’ different situations	4 (2)	1 (0)	30 (13)	158 (70)	33 (15)
17. Planning different resources and teaching methods according to students’ different situations is successful in practice	7 (3)	25 (11)	78 (35)	101 (44)	16 (7)
18. Planning different resources and teaching methods to suit different students’ situation is difficult	6 (3)	43 (6)	32 (33)	118 (55)	24 (3)
19. EFL teachers should undertake reflection within peer groups to address the challenges and problems facing them in their day-to-day professional lives	5 (2)	31 (14)	37 (16)	105 (46)	49 (22)
20. The NCS stresses that EFL teachers should undertake reflection within peer groups to address the challenges and problems facing them in their day-to-day professional lives	2 (1)	2 (1)	16 (7)	155 (68)	52 (23)
21. Undertaking reflection within peer groups to address the challenges and problems facing them in their day-to-day professional lives is successfully achievable in practice	5 (9)	32 (14)	68 (30)	107 (47)	15 (7)
22. Undertaking reflection within peer groups to address the challenges and problems facing them in their day-to-day professional lives is difficult	10 (4)	32 (14)	25 (11)	131 (58)	29 (13)

**TABLE 3:** Teachers’ attitudes toward teacher training for the NCS.

Statements	Strongly disagree	Disagree	Neither agree nor disagree	Agree	Strongly agree
					
	n (%)	n (%)	n (%)	n (%)	n (%)
23. I think the training program I have already had was lecture-based, spoon-fed the trainees, and lacked interaction	7 (3)	32 (14)	38 (17)	132 (58)	18 (8)
24. I think the training program I have already had was short intensive training	8 (3)	15 (7)	58 (25)	126 (56)	20 (9)
25. I think the training program I have already had was attended by teachers on a selective basis and did not cater for all teachers	4 (2)	25 (11)	32 (14)	109 (48)	57 (25)
26. I think the training program I have already had was closely linked to my actual situation of teaching in practice	7 (3)	36 (16)	61 (27)	100 (44)	22 (10)
27. The training I have already had for the NCS has helped me to develop my subject knowledge	6 (3)	10 (4)	72 (32)	120 (53)	19 (8)
28. The training I have already had for the NCS has helped me to improve my teaching practice	3 (1)	22 (9)	66 (29)	123 (54)	15 (7)

**TABLE 4:** The characteristics of the questionnaire respondents (*n* = 227).

Statistics	Location			Gender		Education qualification			English teaching experience (years)					Training experience		
	City	Suburb	Rural	Female	Male	Junior college	Bachelor	Master	<5	6–10	11–15	16–25	>26	Enough	Not enough	No
*n*	80	70	77	205	22	38	173	16	53	57	87	17	13	36	105	86
%	35	31	34	90	10	17	76	7	23	25	38	8	6	16	46	38

The analysis of the data from the questionnaires began once the questionnaires were gathered and compiled. Participants were asked to choose from 1 “strongly disagree” to 5 “strongly agree” in response to each of 28 statements which represented an aspect of teachers’ understandings and beliefs. The analysis of these responses was carried out using the Statistical Package for Social Sciences (SPSS) software. The validity of the questionnaire was achieved through the use of the expert validation method and peer debriefing (Lincoln and Guba, 1985) by asking a British professor and two colleagues to check the face and content validity. Face validity in this case refers not to what the questionnaire actually measured, but to what it superficially appeared to measure. This study was only concerned with how the questionnaire appeared to the subject users—i.e., whether to the ordinary person it looked as if it measured what it was supposed to—not the essential matter of what it really measured. The content validity of the questionnaire was evaluated by getting expert colleagues to assess whether the content of the questions reflected the intended variable or not. The comments from the expert and colleagues went a long way to ensure both the high face and content validity of the questionnaire.

After the questionnaire, in order to capture deeper insights into teachers’ perceptions and ideas and to explore teachers’ conceptions of teaching (Munn and Drever, 2004), we conducted semi-structured face-to-face interviews in Mandarin Chinese with 18 volunteer teachers. A stratified sampling strategy was used to select the interviewees from 92 volunteers (Table 5). Conceptions and beliefs may be implicit and tacitly held, and thus, they cannot always be determined by asking direct questions. Therefore, the interview employed a range of strategies and tools including scenarios and vignettes to elicit teachers’ “inner” views, attitudes, and beliefs from comments and responses on stories depicting scenarios, individuals, and situations (Hughes and Huby, 2012). Although there is not much literature about the use of vignettes, especially within qualitative research or as a complementary method with other data collection techniques, researchers (e.g., Finch, 1987) offer similar descriptions of their use of vignettes in their research. Finch (1987, p. 105) for example, describes vignettes as “short stories about hypothetical characters in specified circumstances, to whose situation the interviewee is invited to respond.” In other words, short scenarios in written or pictorial form are used to elicit participants’ comments or opinions on examples of people and their behavior. An example, of a vignette involving Mr. Lin is appended (Appendix). This is a description of a fairly traditional teacher-centered class. After reading this vignette, participants were asked about their feelings and comments on the vignette and of Mr. Lin’s views and actions. The interview questions were pilot tested with three teachers before embarking upon the study, to discover the appropriate probing and prompting techniques. With the permission of the participants, all the interviews were audio-taped, transcribed, and then analyzed by using thematic coding.

**TABLE 5:** The characteristics of the interview respondents (*n* = 18).

Statistics	Location			Gender		Education qualification			English teaching experience (years)					Training experience		
	City	Suburb	Rural	Female	Male	Junior college	Bachelor	Master	<5	6–10	11–15	16–25	>26	Enough	Not enough	No
Total number in the city	327 (33%)	307 (31%)	356 (31%)	881 (89%)	109 (11%)	218 (22%)	704 (71%)	78 (7%)	188 (19%)	277 (28%)	386 (44%)	109 (11%)	30 (4%)	59 (6%)	515 (52%)	416 (42%)
Number in interview	6 (33%)	5 (31%)	7 (36%)	16 (89%)	2 (11%)	4 (22%)	13 (71%)	1 (7%)	3 (19%)	5 (28%)	7 (39%)	2 (10%)	1 (4%)	1 (6%)	9 (52%)	8 (42%)

The researchers adopted a descriptive-interpretative analysis approach to analyzing the data collected from interviews, that is, descriptive, recognizing that some interpretation was necessary in the analytical process (Maykut and Morehouse, 2002). The codes used to classify the data were, therefore, generated from the data. The researchers stayed focused on the answers to the research question while reading the transcripts and also made allowances for issues or concepts that emerged progressively. Because the NCS was a new curriculum with limited evidence thus far available, the whole analyzing process was based entirely upon the transcripts rather than preconceptions or pre-established codes (Braun and Clarke, 2006). During the coding process, the themes which had the potential to answer the research questions emerged. When new categories emerged, the researchers revisited the previously coded transcripts to identify any instances of the newly emerging categories that had not been noticed in the initial coding. To ensure the validity of this study, the researchers analyzed the data independently, followed by discussions, and then constructed the final set of themes, i.e., teachers’ knowledge of curriculum content; teachers’ understanding of their new roles and the demands of teaching the NCS; and teachers’ views of training for the NCS.

## Findings

The relevant results from the questionnaires and interviews are presented together, to emphasize the complex relationship between “knowing the curriculum” and “understanding the implications for the teacher’s role.” Indeed, it was obvious that most of the participants saw this research as an opportunity to have a voice about improving the quality of EFL teaching and learning and to solve the problems they had in their teaching practices.

### Teachers’ Knowledge of Curriculum Content

In the questionnaire, 98% (223) respondents reported that they were teaching the NCS. All the interviewees had previously taught the 2003 curriculum, and were teaching the NCS when they were interviewed. When all the 18 interviewees were asked, “how do you find the NCS different from the earlier curriculum?” nine teachers said that they had not noticed any differences. The responses were varied. The major differences identified by those who did identify differences were changes in what the curriculum demanded that students learn. Five respondents said that the biggest difference was that the NCS has reduced the learning requirements for students. Two noted the NCS had reduced the number of vocabulary items students were required to learn, and four mentioned that the new textbooks contained less lesson content. These responses recognized change in the textbooks and word lists, but did not recognize the implications, which were that the content of textbooks was expected to play a smaller role in lessons and teachers were intended to supplement the textbook content and vocabulary in ways to best suit the needs of students.

### How English-as-Foreign-Language Teachers Understood Their New Roles and the Demands of Teaching the New Curriculum Standards

#### Understanding Humanistic Values in the New Curriculum Standards

At the heart of the NCS is the renewed emphasis on humanistic values, an idea clearly defined in the curriculum in relation to EFL teaching as teachers considering students’ concerns and feelings, in order to inspire students to form positive attitudes to learning and develop a healthy personality (Ministry of Education [MOE], 2018).

Table 1 shows the agreement levels of respondents to items on this topic. The results suggest the vast majority of participating teachers knew about the proposed development of these values in English language teaching (77%), although only 56% believed that their teaching of humanistic development was successfully achievable in practice, and 68% agreed it was difficult. General speaking, most respondents recognized the requirements of humanistic values in the NCS.

The interview results offer some insights into how teachers understood *humanistic values* as an important and new emphasis in the curriculum. The comments suggested that they linked the idea to a wide range of changes to teaching and learning. Of the 18 respondents, five said that the humanistic value of the English course meant stimulating students’ interest in learning English.

Putting more emphasis on humanistic values is definitely good for students’ practical use. Under the previous earlier curriculum, we were so concerned with teaching grammar and “teaching to the test” that we had little time to dedicate to “speaking.” But now, the NCS encourages learning in happiness and using English in authentic contexts (T4).

However, not all of the respondents showed a positive attitude to the introduction of humanistic values into language teaching and twelve claimed they were unconvinced of the benefits underpinning this approach. Three of them identified humanistic values in the English course as meaning an emphasis on western culture and two said that the humanistic values included promoting humanities exchanges (cultural and educational exchanges). It was notable that many of the comments discussed the changes to textbooks.

Under the earlier curriculum, we did not teach students so much humanistic knowledge because the textbooks include very limited information about this. But now, the new textbooks include more humanistic knowledge, so we put more emphasis on humanistic education (T5).

The interviews suggested the idea of humanistic values was partially understood, remained challenging but that teachers supported the development of this approach.

#### Understanding the Requirements of the New Curriculum Standards (Student-Centered Teaching and Differentiation)

The questionnaire asked teachers to indicate levels of agreement to a number of statements about student-centered teaching. Table 2 shows 88% of the teachers agreed that classes should not be dominated by teachers and should be student-centered, but only around half (52%) believing this was successful in their teaching practice, and 46% claimed it was difficult.

Respondents agreed (89%) that they should set teaching objectives to suit the students’ learning needs and that the curriculum mandated this (88%), although only 74% believed this was successful in practice and 62% agreed it was difficult.

In terms of planning resources, which might be expected to be challenging for teachers who had relied on textbooks in the past, 86% agreed that teachers should plan resources to suit students’ needs, and that the curriculum mandated this, although only 51% agreed this was successful in practice and 58% said it was difficult.

The interview findings revealed more about EFL teachers’ views about student-centered teaching and a traditional, class dominating teacher role. One activity in the interview was to comment on a teaching vignette, where Mr. Lin, a “traditional” teacher, dominated the class. Of the 18 teachers, 16 said that they thought that Mr. Lin used a mainly teacher-centered teaching method, the traditional teaching method in the Chinese teaching context and ten of them said that they used similar teaching methods in their classes.

I like Mr. Lin’s teaching method of reviewing the content at the start of the class, and always ask students questions to make them concentrate. This is very similar to my teaching style in class (T3).

However, the picture was far from uniform. Eight teachers said that they did not agree with Mr. Lin’s view of his role in the English classroom. They thought good teaching should be student-centered and EFL teachers should be organizers. For example,

I do not agree with Mr. Lin. His teaching is teacher-centered. I think a good teacher is not simply an explainer but also has other roles. The most important role is to organize the contents well and let students learn. Students should be the center of the class (T2).

There were some mixed responses where teachers discussed their own changes in practice. Five teachers said that they did not use the same teaching method as Mr. Lin but were more student-centered and used more communicative activities in class. Seven of the teachers reported that they taught very traditionally but also used some communicative activities because they said this presented good teaching. Four teachers’ comments showed that they were strongly affected by the perpetuation of the examination-oriented culture in the Chinese education context. They stated that behavior and attitudes were heavily influenced by their school head teacher’s focusing on exam results. They also pointed out that the evaluation of teachers’ performance was also based on their students’ exam results, which forced teachers to teach for examination purposes rather than adopt a role as organizer.

I understand that students ought to be the center of the class. However, this is demanding and the new approach takes lot of time and energy and cannot help my students to pass examinations. The role as an organizer of differentiated learning may not serve the examination purpose (T7).

The interview and questionnaire results suggested that the emphasis on student-centered teaching and differentiated learning in the NCS was something most teachers knew about, but which they saw as difficult and which they feared may not secure the desired examination outcomes.

#### Teachers’ Views About Undertaking Reflections Within an Inquiry-Led Community

This project asked questions about how teachers learnt about the NCS and about their reflections about their teaching. Four teachers said that they often reflected on and summarized their teaching experiences after their English lessons. Thirteen teachers said that they did this only occasionally. Seven mentioned that they knew that reflection was helpful for improving teaching quality and students’ performance, but only did it where a problem, like poor examination results, was identified.

I sometimes do reflection. Our school requires us to always reflect but we do not follow this suggestion. When students get disappointing exam scores, I discuss them my colleagues within the Teaching and Research group (TRG). I do not have enough energy and time to reflect after each lesson (T2).

The comments from teachers indicated that the activities they undertook included a great deal of reflection on their teaching, although they did not consider these to be reflections because they were not in a writing format. It was clear that this was a challenging area of practice for teachers, reinforcing the questionnaire results (Table 2) in which 91% agreed that “the NCS stresses that EFL teachers should undertake reflection within peer groups to address the challenges and problems facing them in their day-to-day professional lives” but only half (54%) agreed that “undertaking reflection within peer groups to address the challenges and problems facing them in their day-to-day professional lives is successfully achievable in practice.”

The majority of the respondents (71%) agreed that “undertaking reflection within peer groups to address the challenges and problems facing them in their day-to-day professional lives is difficult” which indicates that the they had some difficulty in implementing reflections within an inquiry-led community.

### Teachers’ Views of Training for the New Curriculum Standards

Training is not uniform in its effect and different teachers may perceive it differently. The findings from the questionnaires (Table 3) indicated that the training program teachers had received was “lecture-based, spoon fed, and lacks interaction” (66%), “short intensive” (65%) and “attended by teachers on a selective basis” (73%). Most of the teachers interviewed claimed that they did not get adequate training (17). Seven of the interviewees admitted that they had not learnt the NCS well and thus “did not implement the idea of humanistic values or any new roles, new changes in own professional practice” because they had “no sufficient guidance.”

However, some of the comments about the training they had received suggested it was not designed to promote teacher autonomy or alert them to the implications of the proposed changes, but, instead, focused on the detailed content of the curriculum document:

Our TRG organizes the training for us. The director asked all the EFL teachers to sit down together and read the NCS content aloud by taking turns. So, we are learning the document together (T18).

This technique of reading the curriculum aloud could be a memorization technique, but it is a very particular view of what it means to understand the curriculum. This suggests that policy makers, trainers, and the heads of TRGs to think about the approach to training if it is to be focused on the particular needs of the teachers or the wider implications of the new teacher roles as proposed by the NCS.

T14 shared his difficulties in getting to grips with the changes. He felt that without training he was “experiencing trouble in understanding the new concepts in the NCS” and thus he “had no choice but fall back on previous teaching experiences.” Teachers in this study felt able to confess that they did not understand the NCS but did not recognize many strategies for finding out changes themselves.

All 18 interviewees were asked “what training you would like to have?” They expected more interactive training with real teaching cases (7) and classroom observation with experts’ feedback (9) the most. As T12 mentioned, “by observing experts or successful teachers’ EFL classes, teachers could learn how to solve practical teaching problems and how to implement the NCS in the suggested and effective way.” The interview results suggested that the emphasis in the training “was on the content of the curriculum” (e.g., T9), but had not enabled teachers to understand or address the practical problems they faced.

These teachers are very concerned about their actual teaching practices and wanted more help and support for the substantial issues raised by the NCS – they wanted to relate the training to their own teaching practices.

## Discussion

As we argued earlier, teachers’ understandings of a change and their background training have a significant impact on the implementation of an educational reform (Kırkgöz, 2008; Vähäsantanen, 2015; Tao and Gao, 2017). We outlined in our review of relevant literature a theoretical framework to explore the issues of clarity, complexity, and context (Fullan, 2007; Kırkgöz, 2008; Vähäsantanen and Eteläpelto, 2009) that teachers face in getting to grips with a new curriculum document and changing their role as teachers. If teachers are to implement an educational reform effectively, they are required to have a shared understanding of the theoretical principles and classroom applications of the changes proposed by the innovation (Vähäsantanen and Eteläpelto, 2009; Fullan, 2012; Yang, 2015; Lei and Medwell, 2020). Within the context of curriculum reform, it is essential to change the way teachers think about key components of the innovation, which is a more complex and implicit change (Fullan, 2012; Vähäsantanen, 2015). This means that the change in teachers’ understandings and beliefs plays a key role in the curriculum reform.

A “Chinese culture of teaching and learning” has been widely reported in the past (Cortazzi and Jin, 1996; Hu, 2002) as teacher-dominated, characterized by undifferentiated learning tasks and student persistence. The analysis of the curriculum presented in this article argues that the NCS offers a significant challenge to this past characterization and reinterprets Confucian values in ways which place heavy demands on teachers. Analysis of the NCS identified these demands as a wider range of teacher strategies, a greater range of choice and decision making by teachers, new types and uses of assessment and a new approach toward students. We argue that the NCS for teaching English in China seeks to change the culture of English teaching in the PRC and transform the role of teachers.

Analysis of the NCS also reveals that being an EFL teacher now involves teachers in the design of the curriculum in new ways and places on them the expectation of professional development and being proactive in learning about changes. This is undoubtedly a challenge and it is to be expected that it will take many years to change the beliefs and practices of such a huge teaching force, even when the goals are expressed in Confucian terms and not the language of CLT, which has been so problematic in China in the past (Cortazzi and Jin, 1996; Hu, 2002).

The results of the empirical part of this study of EFL teachers suggest that most teachers now teaching the NCS recognized changes in the content of the curriculum, when these were presented as statements. Moreover, many of the teachers in this study expressed broad agreement with the aims of the curriculum. However, their interviews suggest a much more complex picture. Half of the teachers interviewed did not recognize where the changes had been made or the implications of the changes for their own practices. Even when teachers did recognize changes, they evaluated changes in terms of their existing practices, such as the use and content of textbooks, and discussed assessment of the NCS as focused on goals they recognized from past curricula – performance in tests of reading and writing. These EFL teachers expressed a tension between doing what was best for their students in terms of examination outcomes and changing their practices to make students more independent learners who could use a wider range of resources. This tension between the longer-term goals of the NCS and the short-term assessment mechanisms has been recognized by many scholars (e.g., Kırkgöz, 2008; Yang, 2015; Mei and Wang, 2018; Zhu, 2018; Lei and Medwell, 2020) and extends to the role of the teacher.

The teachers in this study showed cautious enthusiasm for improving students’ experience and enjoyment of learning English, but the inclusion of humanistic values as a key motivator of students and teachers remained only partially understood. The teachers in this study broadly welcomed student-centered teaching, but saw planning activities to suit the needs of all children as impractical and they were suspicious of the practicality and efficacy of such differentiation. Teachers’ responses showed that their choices of teaching methods were determined by their language learning beliefs and the understanding of the NCS. The NCS specifies not only what is to be taught but also how to teach it – a syllabus with teaching guidance for teachers. However, in being specific, it also aims to empower EFL teachers to offer them more choices and options than they may have been used to in the previous, more textbook-based curriculum. In this way, the NCS is both more prescriptive than the old one, in that it specifies teaching methods, but also much less prescriptive because it gives teachers choices. However, many teachers in this study did not appear to recognize the need to develop their own resources, or go beyond the textbook. If this curriculum is to succeed in its goals of producing a “new teacher” who can teach English in student-centered ways, using authentic differentiated resources and seek out the professional development they need, there is some way to go in changing teachers’ views about their roles.

Finally, despite the strong criticism of the delivery and content of the top-down training in the literature (Ping, 2010) and by some teachers in the present study, most respondents had strong faith in the power of this learning approach. The interviews with teachers suggested that they participate in group activities and training and that they would like “more” training. However, the training they described included knowing the NCS, rather than understanding the implications of it. Moreover, these teachers did not see themselves as responsible for finding out about and understanding new developments in the curriculum- something that may also be a significant change to the culture of education in the PRC. The top-down nature of the reform seemed to have left teachers feeling they were not “owning” but “implementing” the innovations. They did not appear to recognize their opportunities to prompt and influence their own reform practices (Vähäsantanen, 2015). This suggests that teachers, trainers and the heads of TRGs, should review their approach to training and the intentions of that training. There is also a need to explore more informal learning (e.g., reflection and self-study), which leaves more space for teacher agency, professionalism and empowerment (Vähäsantanen and Eteläpelto, 2009; Vähäsantanen, 2015; Yang, 2015; Tao and Gao, 2017).

The findings of this study could help to promote the authority, professionalization and ownership of teachers, and can better serve the goals of educational reform. Although the focus of this study is EFL teachers in the Chinese context, it has broader implications for policy makers, researchers, teacher trainers, heads of TRGs and teachers in similar national contexts. The present research highlights that, from an international perspective, introducing new ideas and practices must consider teachers’ existing understanding and experiences of the curriculum as well as the way in which they understand the purposes of the changes, and should promote a shared understanding of policy intentions. The teachers’ understanding of the roles of cooperative reflection, student-centered, and differentiated learning within EFL teaching in the PRC can lead to similar research with teachers from other subject areas and national backgrounds.

## Implications

The challenges of the NCS for teachers were summarized earlier as challenges of clarity, complexity and context (Fullan, 2007; Kırkgöz, 2008; Vähäsantanen and Eteläpelto, 2009) and these challenges are all closely linked. The teachers in this study seemed to have achieved some clarity in their knowledge of the changes made to the NCS. However, there remained serious challenges in terms of their understanding of the complexity of the changes demanded of them. They have sought to interpret these, in some cases, in terms of their existing context, we would argue, the changes proposed to the NCS are a reflection of a much wider cultural change in the expectations of teaching and learning underpinning the curriculum.

Analysis of the NCS showed that it is not simply about changing teaching materials; it is about very profound changes in the goals, methods of English teaching and huge changes to the role of the teacher. The study demonstrated that teachers were engaged this process of reviewing how to implement the NCS, but not necessarily in understanding the underlying implications. The teachers faced challenges with some fundamental issues such as the shift to student-centered teaching and learning and the new role of the teacher. They also identified difficulties with the tension between the unchanged assessment and the new methods in the NCS. The NCS presents a completely different vision of English teaching, a new, wider definition of the curriculum and a broader role for EFL teachers. It specifies not only what is to be taught but also a broader framework within which it is to be taught and the values behind that teaching. It specifies how students should feel about learning and what teachers’ roles should be – it is a syllabus, but also broader teaching guidance for teachers. This issue of complexity (Fullan, 2001, 2007) was not something all the teachers could engage with.

This study has also identified a lack shared understanding between teachers and the NCS of the word “curriculum” and teachers’ understanding and beliefs about the changes in the NCS. These teachers were finding it difficult to understand the notion of the teacher as professional who is more than a figurehead who simply “knows” and “delivers” the curriculum in the changed new curriculum processes, but the teacher is now regarded as “designing” and “creating” the curriculum for their own students. On this issue, the authors of the curriculum and the teachers do not share clarity of understanding.

This study also exposed clear issues of context with were very important to the teachers and mitigated against the changes which the NCS seems to promote. One issue identified by many of the teachers in this study was the unchanged nature of the assessment system in the context of a curriculum with such a different vision of curriculum and teachers. The emphasis of the NCS is not consistent with what is assessed in the examinations. The tension between the NCS and the examinations shows up most in the areas of speaking and listening, involving students in the curriculum designing process, and more formative assessment. As a result, EFL teachers teach what will be assessed in the examinations (e.g., grammar and vocabulary) rather than teach what is outlined and valued by the NCS. This is a very important context for those teachers, who see success in examinations as highly important. This dissonance between understanding and knowledge could impede teachers’ abilities to implement a change as it was intended (Vähäsantanen and Eteläpelto, 2009; Fullan, 2012).

Underpinning these implications, is the complex nature of Chinese middle school EFL teachers’ beliefs at a time when the NCS innovation meets the Confucian Heritage Culture and their existing teaching practices. This is the cultural context of teaching English in China. The results from the questionnaires and interviews have shown that EFL teachers’ beliefs are complicated. On the one hand, most teachers showed a positive attitude toward many new concepts in the NCS, such as student-centered teaching, and teachers’ new roles. They were supportive of these concepts not only because these are encouraged by the policymakers and school head teachers, but also because these are beneficial to students’ all-round development and communicative ability development. On the other hand, the teachers also hold very traditional beliefs and in practice, tended to teach students in a traditional way, for example, their teaching was teacher-centered, teacher-dominated and textbook-based; their teaching strongly emphasized grammar and vocabulary; teachers focused mainly on examination performance; they tended to show their authority and emphasized the importance of controlling the class. These traditional beliefs have been valued in the Chinese educational context for a very long time (Cortazzi and Jin, 1996; Rao, 1998; Run-Hua, 2006), but other beliefs are affected and constrained by local teaching conditions, such as inadequate teaching resources, students’ low English proficiency, the pressure of knowledge-based assessment (Vähäsantanen, 2015). Holding both new and traditional beliefs simultaneously suggests that some teachers seemed to be flexible, practical and were able to embrace the changes in the NCS and fitted them to their own teaching context so they can choose and create a suitable way to teach English that puts their students in an appropriate place between the local teaching reality and the NCS’ requirements. Others may embrace some of the NCS ideals at a conceptual level, but not see them as practicable, while others did not see any changes, apart from the new textbooks, as being either necessary or desirable.

This study has also shown how great the gap between teachers’ beliefs and practices can be. Despite teachers’ seemingly strong beliefs about the importance of some of the key changes in the NCS (such as student-centered teaching, differentiated learning, reflective teaching), there can be great differences between beliefs and teaching practices of teachers. We have explored the training background of the teachers, but it is unclear how teachers’ beliefs were shaped by their training and, given that many of the teachers shared beliefs in this study, it may be that the theoretical content of most training courses about EFL teaching and learning may be somewhat similar. This was outside the scope of this study, as was the duration, method and intensity of such training, but would be an interesting extension to this study. Curriculum change is complex and it is not simply a matter of changing practices, or even of just changing beliefs.

## Data Availability Statement

The original contributions presented in this study are included in the article/supplementary material, further inquiries can be directed to the corresponding author.

## Ethics Statement

The studies involving human participants were reviewed and approved by the Shanghai Normal University and University of Nottingham. The patients/participants provided their written informed consent to participate in this study.

## Author Contributions

Both authors listed have made a substantial, direct, and intellectual contribution to the work, and approved it for publication.

## Conflict of Interest

The authors declare that the research was conducted in the absence of any commercial or financial relationships that could be construed as a potential conflict of interest.

## Publisher’s Note

All claims expressed in this article are solely those of the authors and do not necessarily represent those of their affiliated organizations, or those of the publisher, the editors and the reviewers. Any product that may be evaluated in this article, or claim that may be made by its manufacturer, is not guaranteed or endorsed by the publisher.

## Appendix: Vignette

Look at the example case talking about what Mr. Lin does in his daily English teaching, how do you feel about it? Please justify your answer.

### The Vignette

Mr. Lin leads his class in an animated way. At the beginning of the class, he asks students some questions that based on the reading task they have done before and his students can give their responses quickly. After the review, he teaches the class new content, he always raises some questions to keep students attentive and listening to what he said.

He sees his role as an initiator, an explainer and a class controller. He thinks students won’t learn English unless the teacher goes over the material in a structured way. He believes it is his duty to teach, to explain, and to show students how to learn English and how to do the task.

He says, “it is more practical to set the same teaching objectives for the whole class. Interactive activities such as group work should not take too much time in class because passing the exams is the final teaching goal and the most important thing for English teaching at school.”

1 What do you think about the teaching approach Mr. Lin adopted? Is there anything else you might do for good teaching?
2 What do you think about the role Mr. Lin played in English class? Is there another role you think necessary for good teaching?
3 What do you think about Mr. Lin’s viewpoint? Why?
